# External validation of a mammographic texture marker for breast cancer risk in a case–control study

**DOI:** 10.1117/1.JMI.7.1.014003

**Published:** 2020-02-12

**Authors:** Chao Wang, Adam R. Brentnall, James Mainprize, Martin Yaffe, Jack Cuzick, Jennifer A. Harvey

**Affiliations:** a Kingston University and St. George’s, University of London, Faculty of Health, Social Care and Education, London, United Kingdom; b Queen Mary University of London, Wolfson Institute of Preventive Medicine, Barts and The London School of Medicine and Dentistry, Centre for Cancer Prevention, London, United Kingdom; c Sunnybrook Research Institute, Sunnybrook Health Sciences Centre, Department of Medical Biophysics, Toronto, Ontario, Canada; d University of Virginia, Health Sciences Center, Department of Radiology and Medical Imaging, Charlottesville, Virginia, United States

**Keywords:** mammography, risk assessment, texture, validation, breast density

## Abstract

**Purpose**: The pattern of dense tissue on a mammogram appears to provide additional information than overall density for risk assessment, but there has been little consistency in measures of texture identified. The purpose of this study is thus to validate a mammographic texture feature developed from a previous study in a new setting.

**Approach**: A case–control study (316 invasive cases and 1339 controls) of women in Virginia, USA was used to validate a mammographic texture feature (MMTEXT) derived in a independent previous study. Analysis of predictive ability was adjusted for age, demographic factors, questionnaire risk factors (combined through the Tyrer-Cuzick model), and optionally BI-RADS breast density. Odds ratios per interquartile range (IQ-OR) in controls were estimated. Subgroup analysis assessed heterogeneity by mode of cancer detection (94 not detected by mammography).

**Results**: MMTEXT was not a significant risk factor at 0.05 level after adjusting for classical risk factors (IQ-OR=1.16, 95%CI 0.92 to 1.46), nor after further adjustment for BI-RADS density (IQ-OR=0.92, 95%CI 0.76 to 1.10). There was weak evidence that MMTEXT was more predictive for cancers that were not detected by mammography (unadjusted for density: IQ-OR=1.46, 95%CI 0.99 to 2.15 versus 1.03, 95%CI 0.79 to 1.35, Phet 0.10; adjusted for density: IQ-OR=1.11, 95%CI 0.70 to 1.77 versus 0.76, 95%CI 0.55 to 1.05, Phet 0.21).

**Conclusions**: MMTEXT is unlikely to be a useful imaging marker for invasive breast cancer risk assessment in women attending mammography screening. Future studies may benefit from a larger sample size to confirm this as well as developing and validating other measures of risk. This negative finding demonstrates the importance of external validation.

## 1 Introduction

Over the past few decades, there has been increasing interest in individual risk assessment for breast cancer.^1^^–^^3^ Motivations for this include the identification of individuals at extremely high risk who would be potential candidates for risk-reducing surgery or preventive therapy;^4^ delineation of populations at moderately enhanced risk who might benefit from enhanced screening;^5^ and more recently, identification of populations at sufficiently low risk as not to require screening or risk management.^6^

Breast cancer has a relatively well established hormonal aetiology, in addition to a growing body of knowledge on genetic risk factors.^7^^,^^8^ Although existing risk models have shown a degree of accuracy in prediction [the area under the receiver operating characteristic curve (AUC) ranges around 0.56 to 0.77 for different predictors], it is clear that there is room for improvement.^9^^,^^10^ One area that offers hope for improved risk assessment is utilization of digital mammographic image features. Mammographic density, which is broadly defined as the amount of radio-opaque tissue, is well known as an important independent risk factor for breast cancer.^11^^–^^13^ Some previous research has tried to improve mammographic density risk assessment by looking at other image features of a mammogram, and computational advances in machine learning are starting to spur more work.^14^^–^^17^ A limitation with much of the literature looking at textural or other features from mammograms has been reproducibility.^18^

This study was performed to validate a previously developed texture marker as a breast cancer risk feature by testing its use in an independent case–control dataset.^19^ The texture marker measures the dispersion of breast density within the mammogram. It was previously found to be associated with breast cancer risk in a case–control study of women in Manchester, UK [odds ratio per standard deviation (SD-OR)=1.36], where a subgroup analysis suggested it was most predictive for interval cancers that were detected between routine screening rounds (SD-OR=2.09).^19^ We refer to the marker as MMTEXT for the rest of this paper. Our primary objective was to assess whether MMTEXT is a risk factor for breast cancer in Virginia, USA, after allowing for other classical risk factors from a questionnaire, and for BI-RADS breast density. The prespecified hypothesis was MMTEXT is a risk factor after adjusting for classical risk factors.

## 2 Methods

### 2.1 Study Design

All women, 18 to 89 years of age, diagnosed with breast cancer for the first time at the University of Virginia (UVa) between 2003 and 2013 who had a digital contralateral mammogram at the time of diagnosis were eligible as cases. Case status (invasive breast cancer) was confirmed through chart review. The average time from mammogram to diagnosis of breast cancer is 6 months. All women without a breast cancer diagnosis but identified as having a digital mammogram at UVa during 2003 to 2008 (the more recent being at most 5 years prior to completing the questionnaire, and also one at least 5 years before the questionnaire) were eligible as controls. To ensure a similar age distribution controls were selected based on frequency matching of current age. Risk factor information at the time of questionnaire was retrospectively collected for cases or controls between May 2012 and December 2013, using a self-reported electronic questionnaire that was administered in breast imaging, breast surgery clinic, or medical oncology clinic as previously described.^20^ Women who were eligible as cases but not seen at UVa in more than two years from initiation of patient recruitment were sent a letter for either survey completion by mail or Internet through an electronic token. Women were excluded if they had breast augmentation, prior contralateral mastectomy, or bilateral breast cancer at the time of initial diagnosis as these may affect breast density measurement.

UVa is a public institution that provides reduced fee health care based on need, such that women with greater burden of disease and low resources are frequently referred for care. Thus some differences between cases and controls were expected because controls would mostly include women attending regular screening provided by a health plan, but cases might not. As a result we included several demographic factors for inclusion as adjustments in the analysis. These were the concentric geographical area surrounding UVa, health insurance, whether the woman had been assessed for financial assistance, ethnicity, education, and body mass index (BMI); age in 5-year groups was also adjusted following the study design. Classic hormonal and reproductive risk factors from the questionnaire were combined for adjustment using 10-year risk from the Tyrer-Cuzick (version 7.02; note this version does not incorporate breast density).^3^ Only women aged 40 to 79 years at mammogram were included in order to reflect risk assessment for women attending screening.

Full field digital mammograms (“for processing”) DICOM files from Senographe 2000D, Senographe DS, and Senographe Essential (GE Healthcare, Chicago, Illinois) and Lorad Selenia and Selenia Dimensions (Hologic, Marlborough, Massachusetts) machines were retrieved. Approximately 80% were from GE machines, and data from Hologic were excluded to assess validation of MMTEXT. The reason is that Hologic machines were not used to train MMTEXT, and MMTEXT was higher in Hologic machine (readers are referred to Sec. 5 for regression analysis results showing the impact of different machines). The native resolution of these was 100  μm for the Senographe systems. MMTEXT only uses cranial caudal (CC) views. BI-RADS density category was obtained from clinical records, which were based on the fourth edition lexicon due to the time frame of the study population.

This case–control study was approved by the institutional review boards at the University of Virginia and Sunnybrook Research Institute. The study was compliant with the Health Insurance Portability and Accountability Act. Patients participating on site gave written consent. Patients participating remotely through electronic media were granted waiver of consent.

### 2.2 Risk Marker

MMTEXT was calculated as previously described.^19^ Briefly, image resolution was first downsized by three factors (16, 32, and 64 using images at the same resolution as previously^19^) leading to three new images for which each pixel had a much larger physical area than the original (respectively, $16^{2}=256$, 1024, and 4096 times greater), and with an “average” intensity in that area. To ensure the resulting images are comparable with potential images with different resolutions, all images were downsized to the same target resolutions as previously.^19^ Pixel intensities within the breast were standardized by histogram equalization into 10 bins so that the darkest 10% of all pixels were in bin 1 and the whitest 10% of pixels are in bin 10. A co-occurrence matrix was obtained to give the proportion $p_{k}(i,j)$ of pixel bin i=1,…,10 next to pixel bin j=1,…,10 (in all eight directions) for downsize factor k=1,2,3 ($\underset{i}{\sum }\underset{j}{\sum }p_{k}(i,j)=1$). MMTEXT was calculated as a weighted summation of the so-called “sum average” $t_{k}=\overset{10}{\underset{i=1}{\sum }}\overset{10}{\underset{j=1}{\sum }}(i+j)p_{k}(i,j)$ for downsize factors k=1, 2, 3. On a standardized scale, where the mean and standard deviation of $t_{k}$ are, respectively, zero and unity for each downsize factor k=1, 2, 3, the weights were 30%, 25%, and 45%, respectively, as earlier.^19^

Code to extract MMTEXT from digital mammograms was written by CW using MATLAB software.^21^ Only JM had access to the mammograms for this study and was blinded to case–control status. JM provided the mammographic texture risk score to ARB for analysis, and a list of mammograms to exclude on the basis of automated quality-control software for the images (e.g., to remove mammograms with a nonstandard view, spot compression).

### 2.3 Statistical Methods

The mean value of MMTEXT from left and right CC views was used for controls, but only the contralateral breast was used for cases to limit bias from a dense area due to cancer. MMTEXT was standardized to unit standard deviation and zero mean in controls. It was assessed as a risk factor after adjustment for differences between cases and controls due to demographic factors, age at mammogram, BMI, estimated risk from the Tyrer-Cuzick model (version 7.02),^3^^,^^22^ and with or without adjustment for BI-RADS breast density. Ten-year Tyrer-Cuzick risk was calculated using age at the mammogram, and age at menopause data input was updated accordingly. Other factors in the model were entered following the questionnaire. The only variables that were not included in the Tyrer-Cuzick risk assessment were prior benign breast disease and hormone replacement therapy use, because they were not available.

Spearman correlation was calculated in controls between MMTEXT and standard prognostic variables: age, BMI, 10-year Tyrer-Cuzick risk, and breast density. A generalized additive model was used to show trend lines for age and BMI using tensor splines in controls;^23^ association between MMTEXT and BI-RADS density was inspected using boxplots.

ORs and likelihood-ratio $\chi^{2}$ statistics for MMTEXT were obtained from a logistic regression model that was adjusted for demographic factors, the logarithm 10-year Tyrer-Cuzick risk and optionally BI-RADS density. To compare MMTEXT risk with categorical BI-RADS density, frequency matching of controls was applied, and likelihood-ratio $\chi^{2}$ trend tests were used. Adjusted receiver operating characteristic curves based on the empirical distribution of errors from linear regression models for density and MMTEXT in controls were used to compute adjusted area under the curves (aAUC),^24^ with nonparametric empirical bootstrap confidence intervals.

Heterogeneity of MMTEXT by age, BMI, Tyrer-Cuzick risk, and density was assessed using adjusted logistic regression interaction $\chi^{2}$ tests. Heterogeneity of MMTEXT by mode of detection (mammography/unknown versus none) was tested using a logistic regression for cases only with specific mode of detection as the outcome, adjusted for demographic factors, Tyrer-Cuzick risk, and optionally BI-RADS density. This subgroup analysis was predefined and tested because of results from the development study.^19^ The model fit and assumed linear effect of MMTEXT in the logistic regression was assessed using a generalized additive model with tensor splines for the predictor.

All analyses were undertaken using statistical software R 3.4.1, and with the boot and mgcv packages.^25^^–^^27^

## 3 Results

**Fig. 1:**
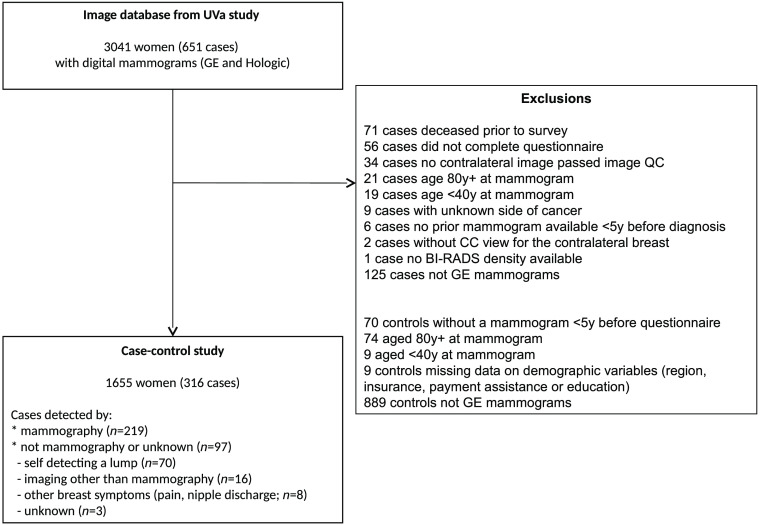
Data flows: sample tested and evaluated for MMTEXT.

The flow of patients is shown in a flow diagram (Fig. 1). Demographic differences were, as expected, apparent between cases and controls (Table 1): cases were more likely to live further away from UVA than controls and be assessed for financial assistance; controls were more likely than cases to have a higher level of education, private health insurance, and be white. Cases were at a higher risk from classical risk factors (Tyrer-Cuzick risk) after adjustment for age and demographic differences (Table 1). It is interesting to note that BMI has a surprisingly higher $\mathrm{LR}-\chi^{2}$ compared with Tyrer-Cuzick risk (Table 1). However, the effect size of BMI was attenuated in a fully adjusted model (IQ-OR 1.08 for BMI versus 1.53 for Tyrer-Cuzick; Table 3).

**Table 1:** Demographic and breast cancer risk factors in cases and controls. For the continuous variables the median and interquartile range (IQR) are given in the case and control columns, with OR for the IQR difference in controls. ORs for demographic factors and age are unadjusted; for risk factors other than age they are adjusted for age and the demographic factors shown.

Factor	Value	Control	Case	OR (95%CI)	$\mathrm{LR}-\chi^{2}$ het
(a) Demographic factors					
Region	Outer	647/1339 (48%)	99/316 (31%)	2.05 (1.58 to 2.66)	30.6 (df=1), P<0.001
Insurance	Yes	927 (69%)	168 (53%)	Ref	27.6 (df=1), P<0.001
	Medicare/aid	387 (29%)	137 (43%)	1.95 (1.51 to 2.52)	
	No	25 (2%)	11 (3%)	2.43 (1.17 to 5.03)	
Financial screening	Yes	50/1339 (4%)	50/316 (16%)	4.85 (3.20 to 7.33)	52.2 (df=1), P<0.001
Education	Less	386/1339 (29%)	146/316 (46%)	2.12 (1.65 to 2.72)	33.9 (df=1), P<0.001
Ethnicity	Not white	96/1339 (7%)	55/316 (17%)	2.73 (1.91 to 3.90)	27.8 (df=1), P<0.001
(b) Classic risk factors					
Age at mammogram	Years	59 (53 to 65)	57 (50 to 65)	0.82 (0.68 to 0.99)	4.3 (df=1), P 0.038
	40 to 44	61 (5%)	24 (8%)	ref	
	45 to 49	143 (11%)	48 (15%)	0.85 (0.48 to 1.52)	
	50 to 54	227 (17%)	57 (18%)	0.64 (0.37 to 1.11)	
	55 to 59	268 (20%)	55 (17%)	0.52 (0.30 to 0.91)	
	60 to 64	249 (19%)	51 (16%)	0.52 (0.30 to 0.91)	
	65 to 69	181 (14%)	30 (9%)	0.42 (0.23 to 0.78)	
	70 to 74	149 (11%)	27 (9%)	0.46 (0.25 to 0.86)	
	75 to 79	61 (5%)	24 (8%)	1.00 (0.51 to 1.95)	
Age at menarche	Years	13 (12 to 14)	12 (12 to 13)	0.82 (0.69 to 0.97)	5.6 (df=1), P 0.018
	Unknown (n)	1	1	NA	
Age first child	<20	112 (8.4%)	46 (14.6%)	1.11 (0.71 to 1.72)	1.2 (df=4), P 0.9
	20 to 29	729 (54.4%)	162 (51.3%)	Ref	
	30+	208 (15.5%)	44 (13.9%)	1.20 (0.80 to 1.78)	
	None	77 (5.8%)	18 (5.7%)	1.16 (0.65 to 2.07)	
	Unknown	213 (15.9%)	46 (14.6%)	1.15 (0.79 to 1.69)	
Menopausal status	Pre	276 (20.6%)	78 (24.7%)	Ref	1.2 (df=2), P 0.5
	Post	1042 (77.8%)	235 (74.4%)	1.01 (0.64 to 1.58)	
	Unknown	21 (1.6%)	3 (0.9%)	0.51 (0.13 to 1.95)	
Age menopause	Years	50 (45 to 52)	49 (42 to 53)	0.89 (0.75 to 1.05)	1.9 (df=1), P 0.17
	Unknown (n)	436	112	NA	
First degree relatives	None	1031 (77.1%)	229 (72.9%)	Ref	4.7 (df=2), P 0.095
	1	286 (21.4%)	77 (24.5%)	1.30 (0.96 to 1.77)	
	2+	21 (1.6%)	8 (2.5%)	2.02 (0.85 to 4.81)	
Height	M	1.63 (1.60 to 1.68)	1.63 (1.57 to 1.68)	1.10 (0.95 to 1.27)	1.5 (df=1), P 0.22
BMI	$\mathrm{kg}/m^{2}$	24.4 (21.9 to 27.5)	26.9 (22.7 to 30.6)	1.40 (1.21 to 1.62)	21.0 (df=1), P<0.001
Tyrer-Cuzick	10 year %	3.14 (2.36 to 4.50)	3.22 (2.27 to 5.02)	1.31 (1.15 to 1.49)	16.8 (df=1), P<0.001

**Fig. 2:**
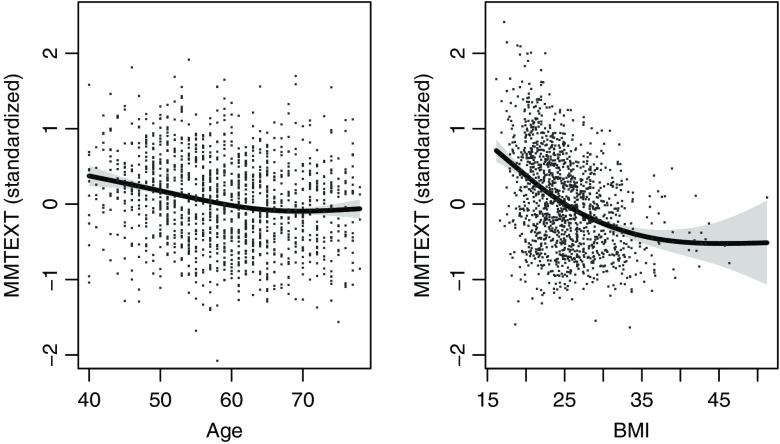
Association between MMTEXT and age and BMI in controls. The points show standardized MMTEXT for each woman. The line in the first plot corresponds to the expected MMTEXT for a woman with average BMI, similarly for the second plot. Standard errors are shaded around each line.

**Fig. 3:**
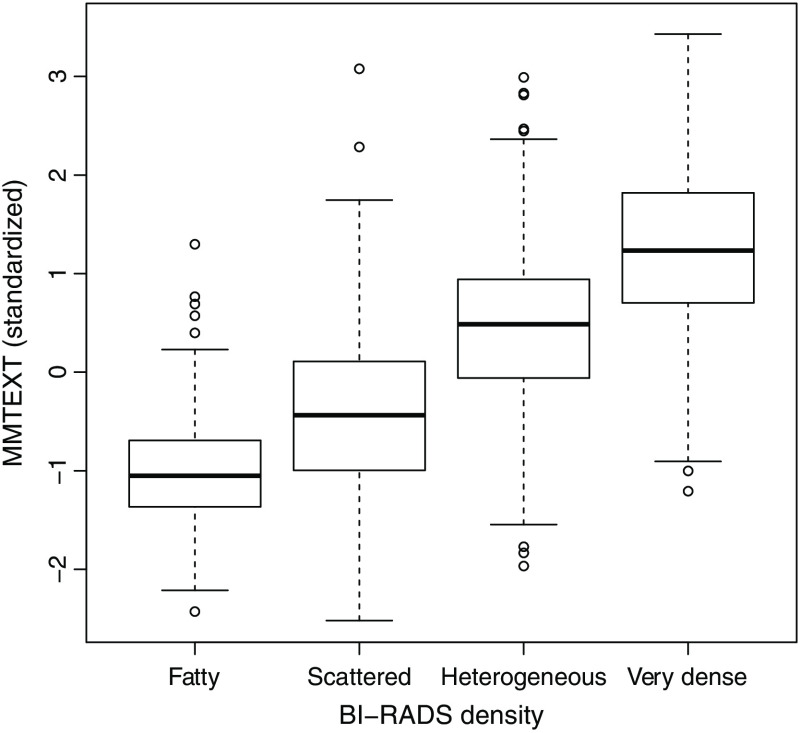
Boxplot distributions of MMTEXT by BIRADS density category.

MMTEXT was negatively correlated with age at mammogram (Spearman correlation ρ=−0.19) and BMI (ρ=−0.37), with an overall nonlinear association shown by Fig. 2. MMTEXT had a small correlation with 10-year risk from the Tyrer-Cuzick model (Spearman ρ=0.07, P=0.007). MMTEXT was strongly positively associated with BI-RADS breast density (Fig. 3, Spearman ρ=0.67).

BI-RADS density was associated with close to a threefold difference in risk between the very dense and fatty categories [OR 2.97 (95%CI 1.58 to 5.57), $\mathrm{LR}-\chi_{1}^{2}$ 15.2 (trend), aAUC 0.54 (95%CI 0.50 to 0.57)] after adjustment for demographic factors and classical risk factors (Table 2). A much less strong risk difference was observed for MMTEXT [OR 1.27 (95%CI 0.72 to 2.24)] based on frequency matching dense and fatty categories in controls, $\mathrm{LR}-\chi_{1}^{2}$ 1.7 (continuous), aAUC 0.50 (95%CI 0.49 to 0.54). On a continuous scale for MMTEXT IQ-OR=1.16 (95%CI 0.92 to 1.46). There was no predictive information in MMTEXT after further adjustment for BI-RADS density [IQ-OR 0.92 (95%CI 0.76 to 1.10), P=0.35]. The fully adjusted logistic regression model fit is shown in Table 3.

**Table 2:** Comparison between adjusted ORs associated with breast density and MMTEXT in the study. OR adjusted for age, BMI, and demographic and classical risk factors. Continuous odds ratios per interquartile range (IQ-OR) in controls are shown in to four categories with the same number of controls as BI-RADS density, for comparison. *Median (IQR).

Factor	Controls (%)	Cases (%)	OR (95%CI)	$\mathrm{LR}-\chi^{2}$ (trend, df=1)
(a) BI-RADS density				
+ Categories				15.2
Fatty	188 (14%)	35 (11.1%)	Ref	
Scattered	545 (40.7%)	126 (39.9%)	1.83 (1.15 to 2.90)	
Heterogeneous	468 (35%)	121 (38.3%)	2.56 (1.56 to 4.19)	
Very dense	138 (10.3%)	34 (10.8%)	2.97 (1.58 to 5.57)	
(b) MMTEXT				
+ Continuous	−0.06 (−0.82 to 0.70)*	−0.08 (−0.78 to 0.75)*	IQ-OR = 1.16 (0.92 to 1.46)	1.7
+ Categories				
1	188 (14%)	50 (15.8%)	Ref	
2	545 (40.7%)	129 (40.8%)	0.81 (0.54 to 1.21)	
3	468 (35%)	105 (33.2%)	0.98 (0.64 to 1.48)	
4	138 (10.3%)	32 (10.1%)	1.27 (0.72 to 2.24)	

**Table 3:** Multivariable logistic regression analysis of breast cancer risk, showing the impact of adding the imaging biomarker MMTEXT to a base model with variables to adjust based on demographic characteristics, classical risk factors, and mammographic density.

Factor	OR (95%CI)	$\mathrm{LR}-\chi^{2}$	P
Base model			
Age (years)		19.7 (df=7)	0.006
(45 to 49)	0.72 (0.38 to 1.37)		
(50 to 54)	0.57 (0.31 to 1.06)		
(55 to 59)	0.50 (0.27 to 0.93)		
(60 to 64)	0.32 (0.16 to 0.63)		
(65 to 69)	0.14 (0.06 to 0.30)		
(70 to 74)	0.12 (0.05 to 0.27)		
(75 to 79)	0.28 (0.12 to 0.66)		
Region		38.7 (df=4)	<0.001
(Outer region 1)	1.40 (0.99 to 1.99)		
(Outer region 2)	1.60 (1.07 to 2.39)		
(Outer region 3)	2.21 (1.45 to 3.36)		
(Outer region 4)	2.70 (1.47 to 4.96)		
Insurance		75.7 (df=2)	<0.001
(Medicare or Medicaid)	4.68 (2.92 to 7.51)		
(None)	0.73 (0.28 to 1.90)		
Financial screening		13.1 (df=1)	<0.001
(Yes)	1.99 (1.07 to 3.70)		
Education		7.0 (df=1)	0.008
(Less)	1.45 (1.06 to 1.99)		
Ethnicity		7.5 (df=1)	0.006
(Not white)	1.69 (1.09 to 2.61)		
Adiposity		17.5 (df=1)	<0.001
(BMI, $\mathrm{kg}/m^{2}$)	1.08 (1.04 to 1.11)		
Classical risk factors		11.6 (df=1)	<0.001
Tyrer-Cuzick (log 10 years)	1.53 (1.16 to 2.01)		
Breast density		17.2 (df=3)	<0.001
(Fatty)	0.53 (0.33 to 0.84)		
(Hetero)	1.50 (1.06 to 2.11)		
(Very dense)	1.84 (1.06 to 3.19)		
New biomarker		0.9 (df=1)	0.35
MMTEXT (per IQR control)	0.92 (0.76 to 1.10)		

Subgroup analysis by mode of cancer detection suggested potential merit of MMTEXT for cancers that were not detected by mammography [unadjusted for density: IQ-OR 1.46 (0.99 to 2.15) versus 1.03 (0.79 to 1.35), Phet 0.10, aAUC 0.55 (95%CI 0.48 to 0.60); adjusted for density: IQ-OR 1.11 (0.70 to 1.77) versus 0.76 (0.55 to 1.05), Phet 0.21; Table 4].

**Table 4:** Logistic regression results for breast cancer risk from MMTEXT for subgroups based on mode of detection.

Factor	Controls (n)	Cases (n)	IQ-OR (95%CI)	$\mathrm{LR}-\chi^{2}$	P
(a) Not adjusted for density					
Mammo detected	1339	222	1.03 (0.79 to 1.35)	0.1	0.8
Not mammo detected	1339	94	1.46 (0.99 to 2.15)	3.6	0.057
Heterogeneity				2.6	0.10
(b) Fully adjusted					
Mammo detected	1339	222	0.76 (0.55 to 1.05)	2.8	0.094
Not mammo detected	1339	94	1.11 (0.70 to 1.77)	0.2	0.6
Heterogeneity				1.6	0.21

Other exploratory analysis found little evidence for heterogeneity of MMTEXT by age, breast density, or Tyrer-Cuzick risk.

## 4 Discussion

This independent study aimed to validate MMTEXT as a risk factor for breast cancer and whether it provides additional information to classical risk factors for risk assessment. Unlike the earlier study,^19^ the estimated IQ-OR=1.16 (0.92 to 1.46) is, however, not statistically significant at 0.05 level. After further adjustment for density the overall predictive ability of MMTEXT was reduced, indicating that much of the information for risk assessment is related to breast density. There was weak evidence that MMTEXT was more predictive for cancers that were not detected mammography (IQ-OR 1.46, 95%CI 0.99 to 2.15); however, after adjustment for breast density, the predictive ability decreases (IQ-OR=1.11, 95%CI 0.70 to 1.77).

This is the first study to assess external validity of MMTEXT and we were able to adjust for classical risk factors using a validated risk model, which is the most important test of a new biomarker.^28^ Image quality was high because full-field digital mammography was used (not scanned film as much previous work^18^) and comparable with the development study.

This study nevertheless differs from the earlier study^19^ in a number of aspects, which might partly explain the different findings. The assessment used a different population than the development sample. A notable difference is that all the cancer cases in this study are invasive, and almost a quarter of cases used for variable selection and model training in the development study were ductal carcinoma *in situ* (DCIS). Invasive cancers differ from DCIS cancers mammographically, as invasive cancers are most often manifest as noncalcified masses so more subtle or occult compared to DCIS cancers.^29^ It is possible that different composition types of cancers affected the results. The demographic factors between the two studies also differ: for example, the percentage of age over 70 years was around 7% in the development study but around 16% in this validation study; the percentage of non-white was also much higher for cases in this study (17% versus 8%).

Although this study failed to find evidence of predictive ability of MMTEXT, BI-RADS was a strong predictor for breast cancer (also in the wider cohort, see Ref. 30). It is arguable that (currently) there is no better measure of mammographic density than visual assessment from an expert,^31^ and in our previous analysis of mammographic density for risk assessment we found that BI-RADS density conferred slightly more predictive information than a volumetric method on the same data.^30^ Another cohort study has confirmed the association of BI-RADS density with risk in this case–control study, when used in the combination with classic risk factors during a follow-up of 19 years.^20^

As discussed above, our subgroup analysis indicate that there may be potential merit of MMTEXT for cancers that were not detected by mammography. If this can be supported by future study with larger sample, there are potential implications for future clinical value. If true, then potentially MMTEXT might have a role as a marker for risk of interval cancer due to masking from mammography. One area this would be useful is to determine eligibility for supplemental screening modalities, such as ultrasound or magnetic resonance imaging. Inspection of the mathematical formula shows that MMTEXT is associated with breast density because it is maximized when white areas of the image are surrounded by other white areas so that images with breast density widely dispersed on the mammogram will have greater MMTEXT values than those with smaller dense areas. A limitation of visual assessment of breast density is the time and expert resources required. In situations where these are important issues, the fully automatic, objective, and freely available MMTEXT might be considered.^21^ MMTEXT currently requires raw mammographic images as it was developed on such images, which may limit its application. Although the same method^19^ can also be trained on processed images, making it suitable for processed images, a potential barrier is that manufacturers’ proprietary processing algorithms may result in images less comparable between different machines. It would nevertheless be interesting for a future study to apply the algorithm describe previously^19^ and test it on processed images.

There are other limitations of the study include the following. First, controls differed from cases due to geography and other socioeconomic and demographic factors, and we needed to adjust for these differences as far as possible in the analysis. Second, self-reported BMI at the time of questionnaire was used, and we had no validation of the self-reported anthropomorphic measures. This is similar to the development study but is expected to have a minimal impact on the overall findings, because it has been seen elsewhere that self-reported measures are likely to be sufficiently accurate.^32^ Third, there is a possible survivorship bias because some women died with breast cancer before the questionnaire was available. However, this is unlikely to lead to an overstatement the main findings and is more likely to weaken them, because on average the deceased cases will have been diagnosed at a more advanced stage than those alive and since density is associated with later diagnosis (masking), this bias might be expected to attenuate the predictive ability of MMTEXT. Fourth, it was not possible to include cases who did not respond to the request to complete a questionnaire (n=47 aged 40 to 79 years). However, if they had been available then the number of cases would only increase by 10%, and it seems unlikely that nonresponse is associated with mammographic density or MMTEXT other than through the factors adjusted for in the analysis such as age and demographics; this issue is also expected to have minimal impact on the main findings. Finally, both the UVa and earlier Manchester studies were predominantly white women and case–control designs.

In conclusion, data from this study do not support risk assessment for invasive breast cancers using MMTEXT, a fully automatic digital mammographic texture risk factor based on raw (“for processing”) DICOM files. This negative finding demonstrates the importance of external validation. Future studies may focus on developing and validating other measures of risk. MMTEXT may, however, have its potential for cancers not detected primarily due to mammography. Further studies are required to verify this, including longer-term effects in cohort studies.

## 5 Appendix

The regression analysis results after adjustment for different machines are shown in Tables 5 and 6 below. The tables include data from women with GE or Hologic machines; data from Hologic machines were excluded in the primary analysis (see Fig. 1).

**Table 5:** Multivariable logistic regression analysis of breast cancer risk, showing the impact of adding the imaging biomarker MMTEXT to a base model with variables to adjust based on demographic characteristics, classical risk factors, and machine type.

Factor	OR (95%CI)	$\mathrm{LR}-\chi^{2}$	P
Base model			
Age (years)		20.6 (df=7)	0.004
(45 to 49)	0.77 (0.44 to 1.33)		
(50 to 54)	0.70 (0.41 to 1.19)		
(55 to 59 )	0.54 (0.31 to 0.93)		
(60 to 64)	0.45 (0.25 to 0.78)		
(65 to 69)	0.26 (0.13 to 0.49)		
(70 to 74)	0.24 (0.12 to 0.46)		
(75 to 79)	0.48 (0.23 to 0.98)		
Region		47.1 (df=4)	<0.001
(Outer region 1)	1.42 (1.08 to 1.88)		
(Outer region 2)	1.49 (1.07 to 2.08)		
(Outer region 3)	2.04 (1.46 to 2.86)		
(Outer region 4)	3.31 (2.01 to 5.46)		
Insurance		60.1 (df=2)	<0.001
(Medicare or Medicaid)	2.76 (1.92 to 3.98)		
(None)	0.87 (0.44 to 1.73)		
Financial screening		10.3 (df=1)	0.001
(Yes)	1.64 (1.04 to 2.60)		
Education		7.2 (df=1)	0.007
(Less)	1.39 (1.08 to 1.79)		
Ethnicity		4.1 (df=1)	0.042
(Not white)	1.38 (0.99 to 1.93)		
Adiposity		7.8 (df=1)	0.005
(BMI, $\mathrm{kg}/m^{2}$)	1.05 (1.03 to 1.07)		
Classical risk factors		12.5 (df=1)	<0.001
Tyrer-Cuzick (log 10 years)	1.49 (1.19 to 1.85)		
Machine type		36.5 (df=1)	<0.001
(Hologic versus GE)	0.54 (0.41 to 0.70)		
New marker		6.2 (df=1)	0.013
MMTEXT (per IQR control)	1.19 (1.04 to 1.36)		

**Table 6:** Multivariable logistic regression analysis of breast cancer risk, showing the impact of adding the imaging biomarker MMTEXT to a base model with variables to adjust based on demographic characteristics, classical risk factors, machine type, and mammographic density.

Factor	OR (95%CI)	$\mathrm{LR}-\chi^{2}$	P
Base model			
Age (years)		20.6 (df=7)	0.004
(45 to 49)	0.76 (0.44 to 1.32)		
(50 to 54)	0.72 (0.42 to 1.23)		
(55 to 59)	0.56 (0.33 to 0.97)		
(60 to 64)	0.48 (0.28 to 0.85)		
(65 to 69)	0.28 (0.14 to 0.53)		
(70 to 74)	0.26 (0.13 to 0.51)		
(75 to 79)	0.55 (0.27 to 1.14)		
Region		47.1 (df=4)	<0.001
(Outer region 1)	1.42 (1.07 to 1.87)		
(Outer region 2)	1.49 (1.07 to 2.07)		
(Outer region 3)	2.03 (1.45 to 2.85)		
(Outer region 4)	3.27 (1.98 to 5.40)		
Insurance		60.1 (df=2)	<0.001
(Medicare or Medicaid)	2.82 (1.95 to 4.07)		
(none)	0.84 (0.42 to 1.68)		
Financial screening		10.3 (df=1)	0.001
(Yes)	1.73 (1.09 to 2.75)		
Education		7.2 (df=1)	0.007
(Less)	1.41 (1.10 to 1.81)		
Ethnicity		4.1 (df=1)	0.042
(Not white)	1.39 (0.99 to 1.95)		
Adiposity		7.8 (df=1)	0.005
(BMI, $\mathrm{kg}/m^{2}$)	1.06 (1.04 to 1.08)		
Classical risk factors		12.5 (df=1)	<0.001
Tyrer-Cuzick (log 10 years)	1.46 (1.17 to 1.82)		
Machine type		36.5 (df=1)	<0.001
(Hologic versus GE)	0.51 (0.39 to 0.67)		
Breast density		21.4 (df=3)	<0.001
(Fatty)	0.62 (0.44 to 0.86)		
(Hetero)	1.33 (1.00 to 1.77)		
(Very dense)	1.51 (0.94 to 2.43)		
New marker		0.0 (df=1)	0.9
MMTEXT (per IQR control)	1.01 (0.85 to 1.19)		
